# Clinical Predictors of Polyautoimmunity in Autoimmune Liver Diseases: Insights into Disease Complexity

**DOI:** 10.3390/jcm14145143

**Published:** 2025-07-20

**Authors:** Özge Güçbey Türker, Çağdaş Kalkan, Gülden Bilican, Emra Asfuroğlu Kalkan, Ali Atay, İhsan Ateş, İrfan Soykan

**Affiliations:** 1 Department of Internal Medicine, Ankara City Hospital, University of Health Sciences, Ankara 06800, Turkey; emra.kalkan@hotmail.com (E.A.K.); dr.ihsanates@hotmail.com (İ.A.); 2 Department of Gastroenterology, Ankara City Hospital, University of Health Sciences, Ankara 06800, Turkey; cagdas.kalkan@hotmail.com (Ç.K.); draliatay@hotmail.com (A.A.); 3 Department of Gastroenterology, Faculty of Medicine, Gazi University, Ankara 06560, Turkey; 4 Department of Gastroenterology, Faculty of Medicine, Ankara University, Ankara 06230, Turkey; irfansoykan@gmail.com

**Keywords:** autoimmune liver diseases, polyautoimmunity, multi-autoimmune syndrome, autoimmune diseases, autoimmune hepatitis

## Abstract

**Background**: Autoimmune liver diseases (ALDs) are a diverse group of chronic inflammatory disorders. Individuals with a history of one autoimmune disease (AD) are at a substantially increased risk of developing additional autoimmune conditions. Polyautoimmunity has increasingly been recognized as a factor associated with a more complex disease course and poorer long-term outcomes. **Methods**: This retrospective, cross-sectional observational study reviewed medical records of patients diagnosed with ALDs who had been admitted to the gastroenterology clinic. **Results**: A total of 457 patients with ALDs were included. Polyautoimmunity was present in 194 patients (42.5%), and multiple autoimmune syndrome (MAS) was diagnosed in 26 of these patients (5.7%). Serological comparisons revealed that antinuclear antibody (ANA) positivity was significantly more common in the polyautoimmunity group. Only 22.2% of the patients with polyautoimmunity were ANA-negative, compared with 52.9% in those without. An ROC curve analysis was conducted to assess the predictive value of clinical and laboratory variables for polyautoimmunity. Among all the tested parameters, ANA positivity (>+2) had the strongest predictive value (AUC: 0.724). A disease duration longer than 6.5 years followed, with a moderate discriminative capacity (AUC: 0.677). Additionally, lower albumin levels (<3.0 g/dL) and elevated erythrocyte sedimentation rates (ESRs) (>29.5 mm/h) were significantly associated with polyautoimmunity. **Conclusions**: In our cohort, 42.5% of patients had at least one additional autoimmune disorder, highlighting the need for a systemic and comprehensive approach to patient care. Simple and accessible markers—such as ANA titers, disease duration, albumin levels, and ESRs—may help to identify patients at greater risk.

## 1. Introduction

Autoimmune liver diseases (ALDs) are a diverse group of chronic inflammatory disorders, traditionally divided into autoimmune hepatitis (AIH), primary biliary cholangitis (PBC), and primary sclerosing cholangitis (PSC), based on clinical presentation, serologic profiles, and histopathological features [1]. AIH is a relatively rare but clinically diverse chronic liver disease characterized by immune-mediated hepatocellular injury. It primarily affects women and can range from asymptomatic elevations in liver enzymes to acute liver failure. While its pathogenesis is thought to be driven mainly by CD4+ T cells, autoantibodies and elevated IgG levels are common features, reflecting broader immune dysregulation. The diagnosis often requires careful integration of clinical, serological, and histological findings [2]. PBC represents a prototypical autoimmune disease, predominantly affecting women at a ratio of approximately 9:1; it is characterized by the presence of well-defined antibodies against the E2 subunit of the pyruvate dehydrogenase complex and is frequently associated with other autoimmune conditions, including celiac disease, autoimmune thyroid disorders, and systemic lupus erythematosus [3]. PSC is a rare, chronic disease marked by inflammation and fibrosis of the bile ducts, leading to strictures and progressive liver damage; it primarily affects young men and is closely linked to inflammatory bowel disease. Although its cause remains unclear, PSC is considered an autoimmune liver disorder with a strong genetic component. No medical therapy that alters its long-term outcomes has been approved to date, although ursodeoxycholic acid is often used to improve liver enzymes [4]. Although the understanding of their pathogenesis has improved over recent decades, the underlying mechanisms remain only partially defined. Genetic susceptibility, immune dysregulation, epigenetic changes, and environmental factors are thought to interact in complex ways to initiate and perpetuate this disease [5,6,7,8].

Individuals with a history of one autoimmune disease (AD) are at a substantially increased risk of developing additional autoimmune conditions, a phenomenon often referred to as “autoimmune tautology” [9]. This framework introduces two related concepts: polyautoimmunity, defined as the coexistence of two or more ADs in a single individual, and multiple autoimmune syndrome (MAS), where three or more distinct ADs are present [10,11,12]. MAS clearly points out that polyautoimmunity is not just a coincidence; it is an expression of underlying shared pathogenic processes [13]. While polyautoimmunity refers to the presence of two or more autoimmune diseases in the same individual, MAS can be seen as a more advanced state, often bringing additional clinical challenges and emphasizing the need for close, multidisciplinary care.

Polyautoimmunity has increasingly been recognized as a factor associated with a more complex disease course and poorer long-term outcomes [14,15,16,17,18]. However, in everyday clinical practice, identifying cases of polyautoimmunity or MAS remains challenging. Many autoimmune conditions share overlapping symptoms and clinical features, which can obscure the diagnosis and delay appropriate management, particularly among patients with ALDs. Against this backdrop, the present study was undertaken to better define the frequency and clinical characteristics of polyautoimmunity and MAS in individuals with ALDs. In addition, we sought to explore demographic, clinical, and serological factors that might be associated with their occurrence.

## 2. Materials and Methods

We performed a retrospective, cross-sectional observational study at the Department of Gastroenterology, Ankara Bilkent City Hospital. This study was approved by the Clinical Research Ethics Committee of Bilkent City Hospital (approval number: E2-22-1951, dated 8 June 2022). We retrospectively reviewed the medical records of patients diagnosed with ALDs who had been admitted to the gastroenterology clinic between March 2019 and January 2023.

Patients were eligible for inclusion if they were 18 years or older, had a confirmed diagnosis of ALDs, and had been followed clinically for at least one year. Diagnoses of AIH, PSC, and PBC were established according to internationally recognized clinical guidelines. AIH was diagnosed by combining clinical, serological, and histological findings. Key features included elevated IgG levels, positive autoantibodies (such as ANA or smooth muscle antibody), and a typical liver biopsy showing interface hepatitis. Ruling out viral hepatitis and drug-induced liver injury was also essential, as these can mimic AIH. Simplified scoring systems were used to support the diagnosis [19]. PSC was mainly diagnosed via magnetic resonance cholangiopancreatography (MRCP), which shows typical bile duct changes. Liver biopsy was performed only if small-duct PSC or overlap with autoimmune hepatitis was suspected. Testing for antimitochondrial antibodies helped rule out PBC, and IgG4 levels were checked to exclude IgG4-related disease [20]. PBC was usually diagnosed by finding high alkaline phosphatase (ALP) levels together with positivity for the antimitochondrial antibody (AMA). If AMA was negative, certain ANA patterns helped. A liver biopsy was performed only if these antibodies were absent or if another liver disease was suspected. High IgM and symptoms such as fatigue or itching also supported the diagnosis [21].

Demographic characteristics such as age, sex, comorbidities, year of diagnosis, and disease duration were systematically recorded. Data regarding concurrent viral hepatitis infections and available liver biopsy findings were also collected. Laboratory values at the time of diagnosis, prior to the initiation of any treatment, were reviewed, including routine biochemistry, liver function tests, blood counts, inflammatory markers, lipid panels, immunoglobulin levels, and a wide panel of autoantibodies.

In addition, the presence and type of extrahepatic AD were documented for each patient. Polyautoimmunity was defined as the coexistence of one additional autoimmune disease alongside ALDs, whereas MAS was defined as the coexistence of two or more additional autoimmune diseases.

Regarding statistical analysis, comparisons between patients with and without polyautoimmunity were performed using the chi-squared test or Fisher’s exact test for categorical variables, and the independent samples *t*-test or Mann–Whitney U test for continuous variables, depending on the distribution. A receiver operating characteristic (ROC) curve analysis was used to assess the diagnostic performance of albumin, ESR, and ANA. Cut-off values were determined based on the Youden index. Factors associated with polyautoimmunity were further analyzed using univariate and multivariate logistic regression models. Odds ratios (ORs) and 95% confidence intervals (CIs) are reported. A two-sided *p*-value < 0.05 was considered statistically significant. Analyses were conducted using IBM SPSS Statistics version 23 (evaluation version, IBM Corp., Armonk, NY, USA).

## 3. Results

A total of 457 patients with ALDs were included. The majority of the cohort was female (n = 400, 87.5%), and the mean age at diagnosis was 48.2 ± 13.2 years. The mean disease duration was 6.44 ± 4.78 years, ranging from 1 to 27 years.

The distribution of diagnoses was as follows: AIH in 203 patients (44.4%); primary biliary PBC in 212 (46.4%); PSC in 16 (3.5%); and overlap syndromes in 26 patients (5.7%), of whom 23 had AIH–PBC overlap and 3 had AIH–PSC overlap. Baseline characteristics by disease group (AIH, PBC, PSC, overlap) are shown in Supplementary Table S1.

Polyautoimmunity was present in 194 patients (42.5%), and MAS was diagnosed in 26 of these patients (5.7%). The frequency of polyautoimmunity varied across the disease groups. It was found in 41.8% of patients with AIH, 39.1% with PBC, 62.5% with PSC, and all patients with overlap syndromes (100%). MAS was most common among overlap patients (46.2%), followed by PSC (18.8%), AIH (3.9%), and PBC (1.4%). The full baseline characteristics are detailed in Table 1.

Regarding associated autoimmune diseases, Hashimoto’s thyroiditis was the most frequent, present in 28 of 203 patients with AIH (13.8%) and in 30 of 212 patients with PBC (14.2%). However, this distribution did not show a statistically significant difference between groups. In contrast, ulcerative colitis and type 1 diabetes mellitus demonstrated significant associations with ALD subtypes. Ulcerative colitis was observed in 9 of 16 patients with PSC (56.3%) (*p* < 0.001). Type 1 diabetes mellitus was found in 11 of 203 patients with AIH (5.4%) and was absent in the other subgroups (*p* = 0.005). Autoimmune hemolytic anemia was present only in the overlap syndrome group, affecting 2 of 26 patients (7.6%), and was absent in all other subgroups, representing a significant difference (*p* = 0.001). These findings are summarized in Table 2.

When clinical characteristics were compared, patients with polyautoimmunity had a longer disease duration (median 7 years) compared with those without (median 4 years) (*p* < 0.001). They were also slightly older at diagnosis (49.5 ± 13.4 years vs. 46.4 ± 12.8 years, *p* = 0.012).

Serological comparisons revealed that ANA positivity was significantly more common in the polyautoimmunity group. Only 22.2% of patients with polyautoimmunity were ANA-negative, compared with 52.9% in those without. Higher ANA titers (+2 to +4) were notably more frequent in the polyautoimmunity group (*p* < 0.001). Anti-dsDNA positivity was also higher among these patients (*p* < 0.001), further supporting the role of systemic autoantibody production in the development of polyautoimmunity. Detailed results of the autoantibody tests in patients with and without polyautoimmunity are presented in Table 3 and comparisons by disease group are provided in Supplementary Table S2.

Several laboratory parameters showed significant differences between groups. Albumin levels were slightly lower in patients with polyautoimmunity (4.00 g/dL vs. 4.20 g/dL, *p* = 0.001), creatinine levels were lower (0.71 mg/dL vs. 0.77 mg/dL, *p* = 0.002), and ESRs were elevated in this group (28 mm/h vs. 21 mm/h, *p* = 0.006). No significant differences were found in AST and ALT levels (Supplementary Materials Table S3).

**Figure 1:**
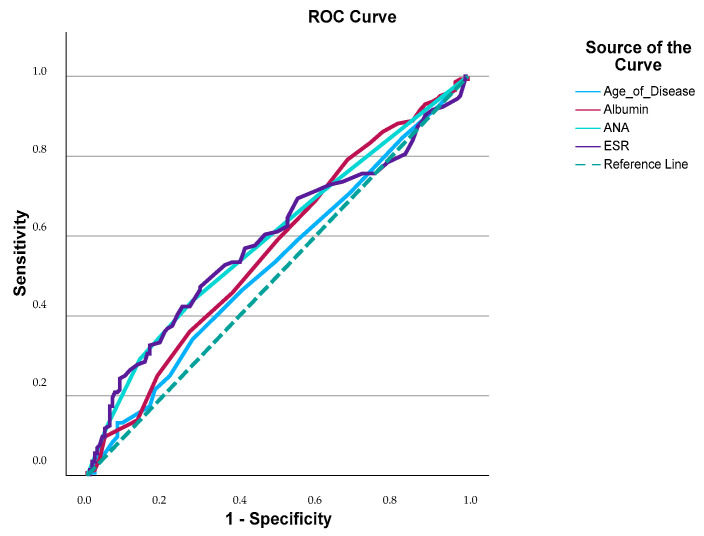
Risk of polyautoimmunity: prediction of disease duration, albumin, ANA, and ESRs using an ROC curve analysis.

An ROC curve analysis was conducted to assess the predictive value of clinical and laboratory variables for polyautoimmunity. Among all the tested parameters, ANA positivity (>+2) had the strongest predictive value (AUC: 0.724). A disease duration longer than 6.5 years followed, with a moderate discriminative capacity (AUC: 0.677). Lower albumin levels (<3.0 g/dL) and elevated ESRs (>29.5 mm/h) were also significantly associated with polyautoimmunity (Supplementary Materials Table S4). However, their individual discriminative performances were relatively modest, with AUCs of 0.594 and 0.591, respectively (Figure 1).

To further clarify these relationships, a multivariate logistic regression analysis was performed. Four independent predictors of polyautoimmunity were identified. Lower albumin levels (OR: 2.348, 95% CI: 1.348–4.103, *p* = 0.003) and ANA positivity (OR: 1.427, 95% CI: 1.163–1.752, *p* = 0.001) remained strong predictors. In addition, longer disease durations (OR: 3.345, 95% CI: 1.231–9.641, *p* = 0.003) and elevated ESRs (OR: 1.021, 95% CI: 1.009–1.034, *p* = 0.001) were independently associated with polyautoimmunity. The univariate and multivariate logistic regression findings regarding factors associated with polyautoimmunity are presented in Table 4.

## 4. Discussion

This study represents one of the largest single-center cohorts evaluating polyautoimmunity among patients with ALDs. While the presence of polyautoimmunity, and even its association with disease severity and genotypic characteristics, has been extensively explored in rheumatologic diseases (e.g., systemic lupus erythematosus and rheumatoid arthritis), this area remains significantly under-investigated in the context of ALDs. Our study addresses this gap by identifying critical clinical and biochemical predictors of polyautoimmunity in this patient population.

Our results demonstrate that 42.5% of patients had at least one additional autoimmune disorder and 5.7% met the criteria for MAS. The frequency of polyautoimmunity observed in our cohort is consistent with the findings from systemic autoimmune conditions such as SLE, with reported prevalence rates ranging between 34.8% and 41% across different studies [8,22,23,24].

The high frequency of polyautoimmunity observed in our cohort brings forward a critical question: Why do autoimmune diseases, whether confined to the liver or involving other organs, tend to cluster within the same individual? Two main hypotheses have been proposed to explain this pattern. One suggests that all autoimmune diseases could be different clinical manifestations of a single underlying systemic disorder, indicating that the distinct conditions we typically classify may, in fact, reflect various expressions of a common immune dysfunction. The second hypothesis is supported by recent genetic studies, which have identified shared susceptibility loci, such as CTLA4, PTPN22, and TNFAIP3, across multiple autoimmune diseases [25]. These emerging models propose that certain autoimmune diseases may share similar etiologies and pathogenic pathways. Numerous studies suggest that polyautoimmunity is not a random occurrence but, rather, a sign of underlying immunological dysregulation [26]. Within this framework, our research emphasizes the crucial need for early diagnosis of polyautoimmunity in clinical settings, particularly in patients with autoimmune liver diseases, as it could imply a more widespread systemic vulnerability to autoimmunity.

Reflecting these shared mechanisms, certain autoimmune diseases also tend to occur together more frequently. In our study, autoimmune thyroid disease was the most common extrahepatic autoimmune condition identified, which is in line with previous findings. For example, Muratori et al. similarly found that thyroid autoimmunity was the most prevalent associated disorder in both AIH and PBC, accounting for more than half of the extrahepatic autoimmune diagnoses within their AIH cohort [27]. The consistent prominence of thyroid autoimmunity across different studies reinforces its potential role as a clinical marker for polyautoimmunity in ALDs and underscores the value of routine endocrine screening in these patients.

Beyond the type of associated autoimmune conditions, certain clinical features also seem to shape the risk of developing polyautoimmunity. In our cohort, patients with polyautoimmunity had a significantly longer disease duration compared with those without, suggesting that persistent immune activation over time may contribute to the emergence of additional autoimmune diseases. They were also diagnosed at an older age, hinting at the possible role of aging-related changes in immune function. These observations align closely with the findings from previous studies, including the RELESSER registry and the Colombian SLE cohort, both of which reported that individuals with polyautoimmunity tend to be older and have a longer history of autoimmune disease compared with those without [8,28]. This pattern has often been linked to the concept of “inflammaging”—the gradual decline in immune tolerance that occurs with aging—combined with cumulative exposure to environmental and epigenetic influences [29]. Together, these factors may create a circumstance in which the immune system becomes more vulnerable, leading to the gradual onset of multiple autoimmune disorders over time. Given the heterogeneous clinical spectrum of ALDs, ranging from asymptomatic to rapidly progressive cases, identifying common predictors of polyautoimmunity remains inherently challenging. Nonetheless, our study aimed to investigate markers such as ANA, ESR, and albumin, which may capture broader immune dysregulation rather than organ-specific inflammation. We reasoned that more severe liver involvement might parallel heightened systemic immune activity, thereby increasing the risk of additional autoimmune manifestations, and conversely, milder disease could indicate less systemic involvement.

ANA is one of the most commonly detected autoantibodies across autoimmune diseases, particularly in type 1 autoimmune hepatitis. However, it is important to recognize that low-titer ANA positivity can also be seen in non-autoimmune conditions, such as chronic hepatitis C [30]. Moreover, about 25% of the healthy population shows ANA positivity at some level, although high-titer ANA is much less common, appearing in only around 2.5% [31]. Despite these limitations in specificity, recent studies have drawn attention to an important nuance: higher ANA titers are more strongly linked to the presence of multiple autoantibodies and an increased risk of systemic autoimmunity [32]. Our findings mirror these observations. We found that polyautoimmunity was more frequently associated with ANA positivity, particularly at higher titers. This suggests that ANA seropositivity—especially when present at higher levels—may act as a valuable marker of broader autoimmune susceptibility. Nonetheless, of note, no single test, including ANA, can definitively identify polyautoimmunity or MAS in patients with ALDs. Autoantibody profiles alone may be misleading due to cross-reactivity or false positives, highlighting the need for a broader clinical and pathological assessment.

Interestingly, in our cohort, some of the laboratory and clinical markers we routinely use to assess liver function or systemic inflammation also showed a strong connection with the presence of polyautoimmunity. One of the standout findings was the erythrocyte sedimentation rate (ESR). As a well-known but nonspecific marker of systemic inflammation, elevated ESR levels have long been linked to autoimmune and autoinflammatory diseases [33]. Consistent with these patterns, patients with polyautoimmunity in our study had significantly higher ESR values compared with those without. This finding supports the hypothesis that chronic, low-grade systemic inflammation may play a favorable role in the development of various autoimmune conditions over time. Persistent inflammatory signals may alter immunological tolerance checkpoints, eventually favoring the development of other ADs.

In addition, we found that lower serum albumin levels were independently associated with polyautoimmunity. Since albumin not only reflects liver synthetic capacity but also acts as an indicator of systemic inflammatory burden, lower levels may signal a broader immune activation reaching beyond the liver. This observation suggests that hypoalbuminemia could serve as a simple yet powerful clue to underlying immune dysregulation in patients with autoimmune liver diseases.

It is also worth noting that traditional markers of liver injury, such as AST, ALT, ALP, GGT, and total bilirubin, were not independently associated with polyautoimmunity in our cohort. Although these parameters varied significantly across different disease subtypes, they did not appear to predict the development of additional autoimmune conditions. This finding highlights an important distinction: polyautoimmunity seems to reflect a broader systemic immunologic environment rather than simply the severity of liver-specific inflammation or injury.

These observations suggest that relying solely on traditional markers of hepatic injury or nonspecific symptoms such as fatigue, abdominal pain, or jaundice, which are common to many hepatic and extrahepatic conditions, may lead clinicians to miss the wider autoimmune landscape that can exist in these patients. A more holistic view, beyond liver-focused parameters, may be crucial for identifying those at greater risk of broader immune dysregulation.

Collectively, these findings suggest that simple and widely available clinical parameters—such as serum albumin, ESR, ANA titers, and disease duration—may serve as practical tools to stratify the risk of polyautoimmunity in patients with autoimmune liver diseases. Incorporating these markers into routine clinical practice could facilitate earlier recognition of concomitant autoimmune diseases, paving the way for more personalized management strategies and potentially improving long-term outcomes.

Nonetheless, several limitations of this study should be acknowledged. The retrospective, single-center design and the relatively short follow-up period of one year may limit the generalizability of the findings and the ability to capture the long-term development of polyautoimmunity. Because this study relies on information recorded in medical charts, it can introduce certain biases and may not always capture data in a consistent or complete way. Our results should therefore be interpreted with this in mind. We also recognize that subclinical or latent polyautoimmunity—cases with autoantibodies or immune markers but no clear symptoms—could not be identified, possibly underestimating the true prevalence. Additionally, liver biopsy remains an important tool for diagnosing ALDs, staging the disease, and assessing liver damage, and future studies should incorporate these features. When it comes to future directions, prospective, multicenter studies with longer follow-up, integrating cytokine profiling and detailed liver histopathology, will be essential to further advance this field.

## 5. Conclusions

The recognition of polyautoimmunity in patients with autoimmune liver diseases carries important clinical implications. In our cohort, nearly half of the patients had at least one additional autoimmune disorder, highlighting the need for a systemic and comprehensive approach to patient care. Simple and accessible markers—such as ANA titers, disease duration, albumin levels, and ESR—may help to identify patients at greater risk. Early detection could support multidisciplinary care and more tailored immunosuppressive strategies, ultimately contributing to improved long-term outcomes.

## Figures and Tables

**Table 1:** Baseline characteristics and demographic and laboratory features of the patients.

	Mean ± std.dev	Median (Min–Max)		Mean ± std.dev	Median (Min–Max)
Age	48.18 ± 13.19	48(18–84)	NEU (×10^9^/L)	4835 ± 3312.29	4100(4.95–34,800)
Age of Disease	6.44 ± 4.78	5(1–27)	MONO (×10^9^/L)	539.25 ± 342.11	500(0–4900)
Creatinin (mg/dL)	0.95 ± 2.78	0.74(0.30–59)	LYM (×10^9^/L)	1955.50 ± 796.4	1900(200–5700)
Protein (g/dL)	7.45 ± 0.83	4.1(0.70–9.70)	SIRI	1890.51 ± 4038.75	1030.51(0–55,440)
Albumin (g/dL)	4.00 ± 0.62	4.10(1.70–5.90)	HB (g/dL)	13.15 ± 1.74	13.30(6.40–17.60)
AST (U/L)	151.91 ± 337.36	35(7–2866)	PLT (×10^9^/L)	270.12 ± 88.01	264(46–734)
ALT (U/L)	170.05 ± 331.70	44(0–2387)	CRP (mg/L)	8.85 ± 13.36	4(0–97)
ALP (U/L)	170.47 ± 148.79	123(0–1013)	T. Cholesterol (mg/dL)	182.4 ± 44.35	113(9–570)
GGT (U/L)	139.55 ± 180.20	69(5–1317)	Triglyceride (mg/dL)	132.18 ± 73.03	47(3–500)
T.BIL (mg/dL)	2.23 ± 4.42	0.76(0.10–32.31)	HDL (mg/dL)	48.43 ± 18.75	47(3–132)
D.BIL (mg/dL)	1.27 ± 3.26	0.20(0–31.61)	LDL (mg/dL)	106.84 ± 51.83	102(10–378)
INR	1.12 ± 0.87	1(0–5)	IGG	16.30 ± 6.41	14.60(5.80–46.30)
Sedimentation(mm/h)	29.48 ± 22.65	24(1–135)	IGM	2.30 ± 1.76	1.68(0.20–46.30)
			IGA	2.69 ± 1.88	2.40(0–24.6)

**Table 2:** Differences between autoimmune liver diseases and other autoimmune diseases.

	AIH (n = 203)	PBC (n = 212)	PSC (n = 16)	Overlap (n = 26)	*p* Value
Hashimoto’s Thyroiditis	28(%13.8)	30(%14.2)	1(%6.3)	3(%11.5)	0.963
Graves’ Disease	0(%0)	5(%2.4)	0(%0)	0(%0)	0.165
Sjögren’s Syndrome	8(%3.9)	10(%4.7)	0(%0)	2(%7.6)	0.603
Systemic Lupus Erythematosus	8(%3.9)	5(%2.4)	0(%0)	1(%3.8)	0.667
Ulcerative Colitis	6(%3.0)	2(%0.9)	9(%56.3)	2(%7.6)	<0.001
Chron’s Disease	2(%1.0)	0(%0)	0(%0)	0(%0)	0.372
Celiac Disease	7(%3.4)	5(%2.4)	0(%0)	1(%3.8)	0.703
Rheumatoid Arthritis	5(%2.5)	5(%2.4)	0(%0)	1(%3.8)	0.785
Ankylosing Spondylitis	2(%1.0)	1(%0.5)	1(%6.3)	1(%3.8)	0.106
Behcet’s Disease	1(%0.5)	1(%0.5)	0(%0)	0(%0)	1.000
Autoimmune Pancreatitis	0(%0)	1(%0.5)	1(%6.3)	0(%0)	0.084
Vitiligo	0(%0)	3(%1.4)	0(%0)	0(%0)	0.438
Familial Mediterranean Fever	2(%1.0)	3(%1.4)	0(%0)	0(%0)	1.000
Multiple Sclerosis	1(%0.5)	2(%0.9)	0(%0)	0(%0)	1.000
Scleroderma	0(%0)	1(%0.5)	0(%0)	0(%0)	1.000
Glomerulonephritis	0(%0)	1(%0.5)	0(%0)	1(%3.8)	0.176
Type 1 Diabetes Mellitus	11(%5.4)	0(%0)	0(%0)	0(%0)	0.005
Addison’s Disease	0(%0)	1(%0.5)	0(%0)	0(%0)	1.000
Mixed Connective Tissue Disease	2(%1.0)	4(%1.9)	0(%0)	0(%0)	0.824
Autoimmune Hemolytic Anemia	0(%0)	0(%0)	0(%0)	2(%7.6)	0.001
Lichen Planus	0(%0)	1(%0.5)	0(%0)	0(%0)	1.000
Psoriasis	0(%0)	2(%0.9)	1(%6.3)	0(%0)	0.083

**Table 3:** Autoantibody tests in the group with and without polyautoimmunity.

Antibody Titer		Polyautoimmunity		*p* Value
		Absent (n = 263) Number (%)	Present (n = 194) Number (%)	
ANA	Negative	139(%52.9)	43(%22.2)	<0.001
	+1 Positive	80(%30.4)	44(%22.7)	
	+2 Positive	22(%8.4)	36(%18.6)	
	+3 Positive	14(%5.3)	53(%22.3)	
	+4 Positive	8(%3)	18(%9.3)	
AMA	Negative	174(%66.2)	116(%59.8)	0.088
	+1 Positive	24(%9.1)	13(%6.7)	
	+2 Positive	34(%12.9)	27(%13.9)	
	+3 Positive	25 (%9.5)	35(%18)	
	+4 Positive	6(%2.3)	3(%1.5)	
ASMA	Negative	239(%90.9)	166(%85.6)	0.179
	+1 Positive	14(%5.3)	14(%7.2)	
	+2 Positive	5(%1.9)	11(%5.7)	
	+3 Positive	4 (%1.5)	3(%1.5)	
	+4 Positive	1(%0.4)	0(%0)	
LKM	Negative	256(%97.3)	186(%95.9)	0.322
	+1 Positive	4(%1.5)	7(%3.6)	
	+2 Positive	3(%1.1)	1(%0.5)	
Anti-Tpo	Negative	251(%95.4)	191(%98.5)	0.280
	Positive	12 (%4,6)	3 (%1.5)	
Anti-Tg	Negative	251(%95.4)	191(%98.5)	0.191
	Positive	12(%4.5)	3(%1.5)	
TRAb	Negative	261(%99.2)	188(%96.9)	0.77
	Positive	2(%0.8)	6(%3.1)	
Celiac Marker	Negative	262(%99.6)	179(%92.3)	<0.001
	Positive	1(%0.4)	15(%7.7)	
Anti-DS DNA	Negative	257(%97.7)	171(%88.1)	<0.001
	Positive	6(%2.3)	23(%11.9)	
ANCA	Negative	252(%95.8)	181(%93.3)	0.289
	Positive	11(%4.2)	13(%6.7)	
APCA	Negative	260(%98.9)	192(%99)	1.000
	Positive	3(%1.1)	2(%1.0)	
RF	Negative	240(%91.3)	146(%75.3)	<0.001
	Positive	23(%8.7)	48(%24.7)	
Anti-CCP	Negative	256(%97.3)	181(%93.3)	<0.001
	Positive	7(%2.7)	13(%6.7)	

**Table 4:** Univariate and multivariate analysis of parameters predicting other autoimmune diseases associated with autoimmune liver disease.

	Univariate Analysis			Multivariate Analysis				
	OR	%95 CI	*p* Değeri	OR	%95 CI	*p* Value	PPV	NPV
Age	0.982	0.968–0.996	0.013					
Gender	1.702	0.941–3.078	0.078					
Disease Duration	1.151	1.100–1.204	<0.001	3.345	1.231–9.641	0.003	0.69	0.74
Creatinin	0.385	0.176–0.840	0.016					
Albumin	1.507	1.100–2.065	0.110	2.348	1.344–4.103	0.003	0.51	0.69
Sedimentation	1.016	1.005–1.027	0.005	1.021	1.009–1.034	0.001	0.60	0.58
ANA	2.049	1.723–2.438	<0.001	1.427	1.163–1.752	0.001	0.71	0.72
Celiac marker	21.955	2.874–167.70	0.003	22.238	2.715–182.17	0.004		
DS DNA	5.761	2.298–14.443	<0.001	3.388	1.208–9.50	0.020		
RF	3.431	2.003–5.875	<0.001	2.197	1.186–4.071	0.012		
CCP	2.627	1.028–6.713	0.044					

## Data Availability

The data presented in this study are available on request from the corresponding author. The data are not publicly available due to privacy and ethical restrictions.

## Supplementary Materials

The following supporting information can be downloaded at: https://www.mdpi.com/article/10.3390/jcm14145143/s1. Table S1: Baseline characteristic, demographic and laboratory features and comparisons of patients. Table S2: Autoimmune tests and comparisons of patients. Table S3: Demographic and laboratory characteristics of the group with and without polyuautoimmunity. Table S4: ROC analysis of significant parameters in polyautoimmunity.
