# Supplementary material for: Clinical Predictors of Polyautoimmunity in Autoimmune Liver Diseases: Insights into Disease Complexity
Source: J Clin Med. 2025 Jul 20;14(14):5143. doi: 10.3390/jcm14145143 (PMC12294987; doi:10.3390/jcm14145143)
Supplement: Supplementary file 1 [file jcm-14-05143-s001.zip › jcm-3674423-supplementary.pdf]

Supplementary Materials:

Table S1: Baseline characteristic, demographic and laboratory features and comparisons of patients

		AIH(n=203)	PBC(n=212)	PSC(n=16)	Overlap(n=26)	P value
Age mean±SD		47.33±15.03	49.69±10.87	39.81±14.18	46.85±12.74	0.017
Gender	Male N(%)	30(%14.8)	18(%8.5)	4(%25)	5(%19.2)	0.059
	Female N(%)	173(%85.2)	194(%91.5)	12(%75)	21(%80.8)	
Age of Disease Median (min-max)		4(1-27)	6(1-21)	7(1-17)	6.5(1-22)	0.134
Viral Hepatitis	Absent N(%)	195(%96.1)	209(%98.6)	16(%100)	25(%96.2)	0.287
	Present N(%)	8(%3.9)	3(%1.4)	0(%0)	1(%3.8)	
Polyautoimmunity	Absent N(%)	118 (% 58.1)	129 (%60.8)	6 (%37.5)	0 (%0)	0.333
	Present N(%)	85 (% 41.9)	83 (% 39.2)	10 (%62.5)	26 (%100)	
Multiple autoimmune syndrome	Absent N(%)	195(%96.1)	209(%98.6)	13(%81.3)	14(%53.8)	0.287
	Present N(%)	8(%3.9)	3(%1.4)	3(%18.8)	12(%46.2)	
Creatinin (mg/dl)		0.72(0.30-59)	0.76(0.41-1.94)	0.72(0.54-1.00)	0.70(0.49-0.99	0.270
Protein (g/dl)		7.5 (5.5-9.70)	7.5 (2.00-9.50)	7.40 (6.80-8.20)	7.40(0.70-8.60)	0.819
Albumin (g/dl)		4(1.7-5)	4.20(2.10-5.90)	4.5(3-4.9)	4.35(2.2-5.30)	<0.001
AST (U/L)		54(7-2866)	30(9-1364)	38.5(16-275)	35(7.0-2866)	<0.001
ALT (U/L)		63(0-2387)	35(7-1317)	60.5(12.9-530)	40.5(11-869)	<0.001
ALP (U/L)		111(0-937)	130(21-1013)	145(53-773)	155(18-417)	0.077
GGT (U/L)		59(5-1081)	66(5-1317)	182(19-658)	105.5(12-571)	0.016
T. BIL (mg/dl)		1.00(0.20-32.31)	0.65(0.10-25)	0.75(0.10-27)	0.90(0.30-19.40)	<0.001
D. BIL (mg/dl)		0.30(0.01-31.61)	0.20(0-13)	0.20(0-18)	0.30(0.10-13)	<0.001
INR		1.10(0.59-15)	1(0-8.20)	1(0.90-1.30)	1(0.80-9.90)	<0.001
Sedimentation (mm/h)		21(1-93)	27(2-111)	22(5-135)	35(3-131)	0.118
NEU (x109/L)		4000(200-34800)	4200(300-27500)	4010(2290-7000)	4560(4.95-19190)	0.701
MONO (x109/L)		470(0-2500)	500(56-4900)	410(300-870)	400(200-1070)	0.078
LYM (x109/L)		1670(200-5700)	2110(300-4400)	1860(1160-2840)	1665(510-4000)	<0.001
Systemic inflammatory response index		1017.44(0-55440)	1033.94(104.3-15921)	983.43(422.7-2360.47)	1302.57(0.67-8591.3)	0.358
HB (g/dL)		13.30(7.4-17.6)	13.40(6.40-17.0)	12.75(8.3-16.6)	13.4(7.40-16.9)	0.801
PLT (x109/L)		257(61-592)	268(46-509)	285.5(171-412)	232.5(134-734)	0.019
CRP (mg/L)		4(0-97)	4.11(0-92)	3(0-48)	4.60(0-29.2)	0.740
Total cholesterol (mg/dl)		174(82-324)	184(38-401)	179(130-222)	174(99-282)	0.060
Triglycerides (mg/dl)		113(22-570)	112.5(9-413)	134.5(48-282)	122.5(53-232)	0.752
HDL cholesterol (mg/dl)		45(3-132)	49(11-118)	53(12-76)	47.5(11-94)	0.046
LDL cholesterol (mg/dl)		97(10-247)	109(10-288)	107.5(5-878)	102(50-192)	0.038
IGG		17.05(6.7-46.3)	13.4(5.80-32.4)	13.25(9.8-21.9)	15.2(7.5-43.5)	<0.001
IGM		1.40(0.4-6.85)	2.5 (0.2-11.9)	1.38(0.4-3.2)	1.65(0.40-4.70)	<0.001
IGA		2.55(0.10-24.6)	2.30(0-8.9)	2.21(0.9-4.4)	2.30(1.06-10.8)	0.090

AIH: Autoimmun Hepatitis, PBC: Primary biliary cholangitis, PSC: Primary sclerosing cholangitis, SD: standard deviation, AST: aspartate aminotransferase ALT: alanine aminotransferase, ALP: alkaline phosphatase GGT: gamma-glutamyl transferase, T.Bil: total bilirubin, D. Bil: direct bilirubin, INR: international normalized ratio, NEU: neutrophil count, MONO: monocytes count, LYM: leukocyte count HB: hemoglobin concentration, PLT: platelet count, CRP: C-reactive protein, IG: Immunoglobulin

Table S2: Autoimmune tests and comparisons of patients

Autoimmun Tests		AIH(n=203)	PBC(n=212)	PSC(n=16)	Overlap(n=26)	p value
ANA	Negative	43(%21.2)	121(%57.1)	9(%56.3)	9(%34.6)	0.015
	+1 Positive	84(%41.4)	27(%12.7)	7(%43.8	6(%23.1)	
	+2 Positive	32(%15.8)	23(%10.8)	0(%0)	3(%11.5)	
	+3 Positive	34(%16.7)	26(%12.3)	0(%0)	7(%26.9)	
	+4 Positive	10(%4.9)	15(%7.1)	0(%0)	1(%3.8)	
AMA	Negative	193(%95.1)	67 (%31.6)	16(%100)	14(%53.8)	0.005
	+1 Positive	1(%0.5)	35(%16.5)	0(%0)	1(%3.8)	
	+2 Positive	5(%2.5)	53(%25)	0(%0)	3(%11.5)	
	+3 Positive	4(%2)	50(%23.6)	0(%0)	6(%23.1)	
	+4 Positive	0(%0)	7(%3.3)	0(%0)	2(%7.7)	
ASMA	Negative	162(%79.8)	210 (%99.1)	14(%87.5)	19(%73.1)	<0.001
	+1 Positive	23(%11.3)	0(%0)	2(%12.5)	3(%11.5)	
	+2 Positive	13(%6.4)	1(%0.5)	0(%0)	2(%7.7)	
	+3 Positive	4(%2)	1(%0.5)	0(%0)	2(%7.7)	
	+4 Positive	1(%0.5)	0(%0)	0(%0)	0(%0)	
LKM	Negative	191(%94.1)	211(%99.5)	16(%100)	24(%92.3)	0.009
	+1 Positive	9(%4.4)	0(%0)	0(%0)	2(%7.7)	
	+2 Positive	3(%1.5)	1(%0.5)	0(%0)	0(%0)	
Anti-Tpo	Negative	175(%86.2)	192(%90.6)	14(%87.5)	21(%80.8)	0.308
	Positive	28 (%13.8)	20(%9.5)	2(%12.6)	5(%19.2)	
Anti-Tg	Negative	191(%94.1)	209(%98.6)	16(%100)	26(%100)	0.219
	Positive	12(%5.9)	3(%1.4)	0(%0)	0(%0)	
TRAb	Negative	202(%99.5)	205(%96.7)	16(%100)	26(%100)	0.220
	Positive	1(%0.5)	7(%3.3)	0(%0)	0(%0)	
Celiac Marker	Negative	196(%96.6)	206(%97.2)	14(%87.5)	25(%96.2)	0.216
	Positive	7(%3.4)	6(%2.8)	2(%12.5)	1(%3.8)	
Anti- DS DNA	Negative	188(%92.6)	205(%96.7)	14(%87.5)	21(%80.8)	0.007
	Positive	15(%7.4)	7(%3.3)	2(%12.5)	5(%19.2)	
ANCA	Negative	192(%94.6)	209(%98.6)	11(%68.8)	21(%80.8)	<0.001
	Positive	11(%5.4	3(%1.4)	5(%31.3)	5(%19.2)	
APCA	Negative	202(%99.5)	209(%98.6)	16(%100)	25(%96.2)	0.348
	Positive	1(%0.5)	3(%1.4)	0(%0)	1(%3.8)	
RF	Negative	170(%83.7)	180(%84.9)	14(%87.5)	22(%84.6)	0.987

	Positive	33(%16.3)	32(%15.1)	2(%12.5)	4(%15.4)	
Anti-CCP	Negative	196(%96.6)	202(%95.3)	15(%93.8)	24(%92.3)	0.459
	Positive	7(%3.4)	10(%4.7)	1(%6.3)	2(%7.7)	

ANA: antinuclear antibody, AMA: anti-mitocondrial antibodies, ASMA: anti-smooth muscle antibodies, LKM: anti-liver/kidney microsomes antibodies, Anti-Tpo:anti-thyroperoxidase antibody, Anti-tg: anti-thyroglobulin antibody TRAb: TSH receptor antibodies, Anti-DS DNA: anti-double-stranded-deoxyribonucleic acid-antibody ANCA: anti-neutrophil cytoplasmic antibody APCA: Anti-parietal cell antibodies, RF: rheumatoid factor, Anti-CCP: anti-citrullinated cyclic peptides antibodies

Table S3: Demographic and laboratory characteristics of the group with and without polyuautoimmunity

			Poliautoimmunity		pvalue
			Absent (n=263)	Present (n=194)	
Age mean±SD			49.51±13.39	46.39±12.75	0.012
Gender	Male	N (%)	39(%14.8)	18(%9.3)	0.076
	Female	N (%)	224(%85.2)	176(%90.7)	
Disease DurationMedian (min-max)			4(1-22)	7(1-27)	<0.001
Viral Hepatitis	Absent	N(%)	254(%96.6)	191(%98.5)	0.215
	Present	N(%)	9(%3.4)	3(%1.5)	
Creatinin (mg/dl)			0.77(0.30-59)	0.71(0.41-1.94)	0.002
Protein (g/dl)			7.40 (2.00-9.20)	7.50(0.70-9.70)	0.123
Albumin (g/dl)			4.20(1.70-5.90)	4.00(2.10-5.00)	0.001
AST (U/L)			35(7.0-2866)	35.50(7.0-1948)	0.981
ALT (U/L)			46(0-2387)	43(7-1537)	0.742
ALP (U/L)			115(0-1013)	130(18-956)	0.156
GGT (U/L)			59(5-1317)	86(5-987)	0.053
T.BIL (mg/dl)			0.80(0.10-32.31)	0.70(0.10-27)	0.270
D.BIL (mg/dl)			0.20(0-31.61)	0.20(0-18)	0.879
INR			1(0-2.5)	1(0.30-15)	0.675
Sedimentation (mm/h)			21(1-135)	28(2-131)	0.006
NEU (x109/L)			4010(300-34800)	4130(4.95-19900)	0.955
MONO (x109/L)			500(79-2500)	480(0-4900)	0.711
LYM (x109/L)			1890(200-5700)	1955(200-4800)	0.181
Systemic inflammatory response index			1056.43(37.5-55440)	981.92(0-17760)	0.285
HB (g/dL)			13.40(6.40-17.60)	13.30(7.40-17.00)	0.754
PLT (x109/L)			261(46-592)	266(67-734)	0.674
CRP (mg/L)			4.40(0-97)	3.85(0-94.8)	0.182
Total cholesterol (mg/dl)			180(38-351)	176(97-401)	0.383
Triglycerides (mg/dl)			113(9-570)	113.5(31-413)	0.698
HDL (mg/dl)			46(3-132)	48(9-99)	0.147
LDL (mg/dl)			102(10-235)	102(17-878)	0.746
IGG			14.85(5.80-41.40)	14.40(7.40-46.30)	0.364
IGM			1.60(0.20-11)	1.76(0.40-11.90)	0.486
IGA			2.40(0-9.30)	2.30(0.10-24.60)	0.289

Table S4:ROC analysis of significant parameters in polyautoimmunity

Tests	Cut-off	AUROC	Sensitivity	Specificty	PPV	NPV	P value
Albumin	<3	0.594	0.69	0.51	0.51	0.69	0.001
		0.541-0.647	0.62-0.75	0.45-0.57	0.46-0.55	0.64-0.73	
Sedimentation	>29.5	0.591	0.47	0.71	0.60	0.58	0.006
		0.526-0.656	0.39-0.55	0.63-0.77	0.54-0.66	0.52-0.64	
ANA	>2	0.724	0.55	0.83	0.71	0.72	<0.001
		0.676-772	0.48-0.62	0.78-0.87	0.66-0.75	0.67-0.76	
Disease duration	>6.5	0.677	0.58	0.69	0.58	0.69	<0.001
		0.627-0.727	0.51-0.65	0.63-0.75	0.53-0.63	0.65-0.74	
